# Family, social, and built environment: examining associations with physical activity and sedentary time in adolescents

**DOI:** 10.3389/fspor.2026.1709140

**Published:** 2026-02-19

**Authors:** Mónica Suárez-Reyes, Alyssa M. Button, Robbie A. Beyl, Peter T. Katzmarzyk, Amanda E. Staiano

**Affiliations:** 1 Pennington Biomedical Research Center, Baton Rouge, LA, United States; 2 Escuela de Ciencias de la Actividad Física, el Deporte y la Salud, Universidad de Santiago de Chile, Santiago, Chile; 3 Virginia Commonwealth University, Richmond, VA, United States

**Keywords:** family support, health behaviors, physical behaviors, sedentary behaviors, youth lifestyle

## Abstract

**Introduction:**

Physical activity and sedentary time, along with other health-related behaviors, are influenced by various environmental factors. This study examines the associations of family, social, and built environments with physical activity and sedentary time in adolescents.

**Methods:**

Participants aged 10–16 years were enrolled, with data from 308 adolescents analyzed. Physical activity and sedentary time were assessed using accelerometers, while family, social, and built environments were evaluated through questionnaires completed by parents/guardians. General Linear Models were used to examine associations between environmental factors and physical activity and sedentary time, adjusting for covariates.

**Results:**

On average, participants engaged in 27.7 min of moderate-to-vigorous physical activity, 232.8 min of light physical activity, took 6,800 steps, and spent 595.4 minutes in sedentary time per day, with only 10% meeting the recommended 60 min of moderate-to-vigorous physical activity. Higher family support was associated with more moderate-to-vigorous physical activity (*β* = 5.2), more light physical activity (*β* = 7.9), more steps per day (*β* = 688), and less sedentary time (*β* = −13.2) per additional unit of family support. The social environment total score was not associated with physical activity or sedentary time. However, when analyzing individual components, social cohesion was negatively associated with physical activity and positively associated with sedentary time, while social control showed no significant relationship. No associations were found between the built environment and physical activity or sedentary time.

**Conclusions:**

This study highlights the significant role of family support in promoting physical activity and reducing sedentary time in adolescents, with the family environment showing the strongest associations. In contrast, the social and built environments had limited influence on physical activity behaviors. The findings underscore the importance of family-based strategies to encourage active lifestyles.

**Clinical Trials Registration:**

# NCT02784509.

## 1 Introduction

Substantial evidence highlights the association between physical activity (PA), particularly moderate-to-vigorous physical activity (MVPA), and positive health outcomes during childhood and adolescence (1, 2). On the other hand, growing research on time spent in sedentary behaviors (sedentary time) suggests a potential negative relationship with health outcomes at these ages (3). Current public health guidelines emphasize the need for young people to engage in regular physical activity and limit sedentary time to support their overall health and well-being. Despite the recommendations, more than 80% of young people aged 11–17 years fail to meet the physical activity guidelines, indicating significant challenges in promoting active lifestyles (4). Building on the ecological model, it is recognized that, like other health-related behaviors, physical activity and sedentary time are shaped by a range of environmental factors (5). Given that young people have less autonomy over their health-related choices, the influence of contextual factors such as family, social, and built environment may differ from what is observed in adults (6).

The family is the primary environment where habits, values, and beliefs related to physical activity and sedentary behaviors are cultivated (7). Parents and other family members can support children's physical activity in various ways. This support may be instrumental, such as providing transportation, equipment, or supervision; or emotional, through encouragement, praise; or co-participation in activities (8). Family support has been associated with higher levels of PA and lower levels of sedentary time in youth populations, as well as greater engagement in various types of PA (9). Morrissey et al. (7) reported moderate positive associations (*r* = 0.32–0.53) between family support and MVPA in adolescents (13–17 years), with greater family support linked to higher levels of MVPA (*β* = 0.66). This effect appeared to be more pronounced in girls than in boys. In a systematic review and meta-analysis, Laird et al. (8) reported small but significant associations between family support and PA in girls. Similarly, Dowda et al. (10) observed positive associations between family or parental support and PA in a study of 5th- and 7th-grade children. Interestingly, this association was evident only when PA was self-reported by the children (*β* = 0.23) and their parents (*β* = 0.41). However, no association was observed when MVPA was measured objectively using accelerometers. Other studies have reported variable associations between family support and children's PA. For example, a systematic review by Rhodes et al. (11) found that parental support was negatively associated with child age, indicating that older children and youth were less likely to receive parental support. A meta-analysis by Yao et al. (12) reported that parental support was positively associated with child PA, with a medium effect size highlighting its importance as a correlate of child PA (11, 12). While some argue that forms of support requiring greater parental involvement, such as co-participation or modeling, have a stronger impact on children's PA (13, 14), others have found stronger associations with emotional support, such as encouragement and praise (12). The variability in the methods used to measure PA and the types of family support provided are important factors to consider. However, the small to moderate strength of the associations reported in most studies suggests that family support accounts for only a limited amount of the observed variability in PA behaviors. This underscores the need to explore other environmental factors that influence PA and sedentary time in young people.

Beyond the family, the neighborhood social environment has been widely studied for its role in shaping PA and time spent in sedentary behaviors among children and adolescents (15). Collective efficacy is an indicator of the social environment. Collective efficacy is defined as the strength of relationships among community members and their shared ability to address safety concerns, fosters a supportive and secure environment that encourages PA, particularly for younger populations (16) Some studies have shown that a positive neighborhood social environment, characterized by greater collective efficacy, is associated with higher PA levels, based on parental reports in both studies (17, 18). However, evidence of this relationship remains mixed when other factors are considered. Sullivan et al. (18) found that in high-income countries (e.g., United States, Canada, Australia), collective efficacy is positively associated with PA (*β* = 1.86), whereas in low- and middle-income countries (e.g., Kenya, Brazil, Colombia), the association was negative (*β* = −1.96). The authors attribute these differences to the nature of PA: in wealthier countries, it is primarily optional and leisure-based, whereas in lower-income countries, it may be obligatory, transportation-related, or occupational. These contrasting findings highlight the complex and context-dependent nature of the relationship between social environments and PA across different income settings.

The relationship between the built environment, i.e., the spaces where we live and play, and PA in young people has been widely documented. Evidence suggests that availability and access to spaces such as parks, bicycle paths, sports infrastructure, or safe and walkable neighborhoods may influence PA and sedentary behaviors in children and adolescents. However, the observed associations have been small to moderate and are influenced by factors such as age, the type of environmental component, and the methods used to assess the built environment and PA (19, 20). Dowda et al. (10) observed a positive correlation between MVPA and access to places and resources for PA, with both variables measured objectively. This correlation is somewhat stronger in older children compared to younger children (*r* = 0.21 and *r* = 0.16, respectively), suggesting that as children grow older, they are more likely to engage in PA in locations away from home. Similarly, Prins et al. (21) explored the relationship between the built environment (e.g., access to parks and conditions for active transportation) and adolescent participation in PA. The results indicated that those with greater access to these built resources were 73% more likely to engage in sports activities and 66% more likely to be more active during their free time. However, these associations were only observed when the perceived built environment was considered, with no significant association found when the built environment was assessed objectively. While the link between the built environment and PA has been more extensively studied, the relationship between the built environment and sedentary time in young people has been less explored and more challenging to interpret. Bejarano et al. (22) found that certain built environment features were positively associated with sedentary time, while others correlated with increased overall sedentary time in adolescents. This suggests that some environmental attributes may influence sedentary time differently than PA.

As has been discussed, various factors within the family, social, and built environments may contribute to shaping PA and sedentary time in young people. However, a comprehensive understanding still requires examining these factors collectively, offering a more holistic perspective on how they influence PA patterns in adolescents. Furthermore, exploring these factors together allows for a deeper understanding of the relative importance of each component. The objective of this study is to determine the associations between the family, social, and built environment with PA and sedentary time in adolescents. Such an approach is essential for informing public health strategies and interventions aimed at promoting PA in adolescents.

## 2 Methods

### 2.1 Participants

Participants aged 10–16 years, an age range defined by the World Health Organization as adolescence (10–19 years), were enrolled between 2016 and 2018 in the prospective observational TIGER Kids study (Translational Investigation of Growth and Everyday Routines in Kids; NCT02784509). This analysis, which is cross-sectional in design, used baseline data. Adolescents were recruited from Baton Rouge, Louisiana, United States through a variety of convenience sampling methods, including email lists, health fairs, schools, and social media outreach. To be eligible, participants had to be able to follow instructions and complete study activities, not be pregnant, not follow a medically restricted diet, and have no major physical or mental limitations that would prevent movement or use of an accelerometer.

Of the 342 participants initially enrolled, 308 were included in this analysis. Participants were excluded due to missing values for body weight, incomplete questionnaires (family support, social support, and built environment), or lacking valid accelerometer data. The characteristics of the sample are presented in Table 1. The study received approval from the Institutional Review Board at Pennington Biomedical Research Center (IRB #2016-028). Written consent of the parent/guardian and written assent of the adolescent were obtained at the first visit.

**Table 1:** General characteristics of the sample (*n* = 308).

Variable	Median	p25	p75	*n*	%
Age (y)	12.7	11.3	14.4		
Girls				166	53.9
Boys				142	46.1
White				178	57.8
African American				106	34.4
Other race				24	7.8
BMI (kg/m^2^)	22.2	18.6	27.9		
BMI z-score	1.02	−0.04	1.91		
Underweight				9	2.9
Normal weight				145	47.1
Overweight				45	14.6
Obesity				109	35.4
Accelerometer wear time (min/day)	864.9	818.0	904.9		
MVPA (min/day)	27.7	19.9	42.8		
LIPA (min/day)	232.8	196.9	278.2		
Steps (steps/day)	6,896	5,559	8,606		
Sedentary time (min/day)	595.4	535.3	637.1		
Physically active (≥60 min/day MVPA)				31	10.1
Family environment (total score)	2.8	2.0	3.5		
Watch child do PA	2.0	2.0	3.0		
Encourage PA	4.0	3.0	5.0		
Provide transportation	3.0	2.0	4.0		
Do PA with child	2.0	2.0	3.0		
Social environment^a^ (total score)	4.0	3.7	4.3		
Social cohesion	3.8	3.4	4.2		
Social control	4.2	3.8	4.6		
Built environment (z-score)	0.01	−1.64	2.03		
Land use diversity	3.5	2.8	4.5		
Proximity to recreation facilities	3.6	2.9	4.6		
Land access	2.0	1.0	2.5		
Street connectivity	3.0	2.0	3.5		
Walk facilities	2.5	1.0	3.0		
Aesthetic	2.0	1.5	3.0		
Pedestrian safety	2.8	2.3	3.0		
Child safety	2.0	1.4	2.4		

a Social environment or Collective efficacy is obtained from the average of Social Cohesion and Social Control. BMI, body mass index; PA, physical activity; LIPA, light intensity physical activity; MVPA, moderate-to-vigorous physical activity.

### 2.2 Procedures

After receiving written consent from the parent/guardian and written assent from the adolescent, participants were provided with an accelerometer to wear on the hip for seven consecutive days. Following this period, the adolescent, accompanied by their parent/guardian, attended a clinical visit.

During the visit, demographic information was collected from the parent/guardian, and questionnaires assessing family, social, and built environment were administered. Additionally, anthropometric measurements, including height (cm) and weight (kg), were measured following a standardized protocol. Body mass index (BMI in kg/m^2^) was then calculated and converted into percentiles based on the 2020 Centers for Disease Control and Prevention (CDC) Growth Charts for the United States (U.S.) using R version 4.2.3 and the *cdcanthro* package. Overweight was classified as a BMI at or above the 85th percentile and less than the 95th percentile, while obesity was defined as a BMI at or above the 95th percentile.

All surveys were collected and managed using the Research Electronic Data Capture (REDCap) system, a secure, web-based platform hosted by Pennington Biomedical Research Center (23, 24). REDCap supports research data collection with features such as a user-friendly interface for validated data entry, audit trails for tracking data manipulation, automated export functions for statistical analysis, and the ability to integrate external data sources.

### 2.3 Physical activity and sedentary time

Physical activity and sedentary time were assessed using an ActiGraph GT3X + accelerometer (Actigraph, Ft. Walton Beach, Pensacola, FL, USA) worn on an elastic belt at the hip, aligned with the left midaxillary line. Participants were given written and verbal instructions and asked to wear the device continuously (24 h/day), except during water-based activities, for at least 7 days, including 2 weekend days. Sleep time was identified using the Tudor-Locke algorithm (25), while non-wear time was determined as intervals of at least 20 consecutive minutes of zero activity intensity counts. Only days with a minimum of 10 h of awake wear time were included in the analysis, and participants had to have at least four valid days, one of which had to be a weekend day. Sedentary time was classified as activity registering fewer than 25 counts per 15 s, while moderate-to-vigorous physical activity (MVPA) was marked by 574 counts or more per 15 s (26). Light intensity physical activity (LIPA) was calculated by subtracting the time spent sedentary and in MVPA from the total awake wear time. The steps per day were also considered as a PA indicator. Participants who averaged 60 min of MVPA per day or more across valid days were classified as physically active based on the World Health Organization (WHO) guidelines (27).

### 2.4 Family, social, and built environment

The parent/guardian completed a series of surveys, including assessments of the family, social, and built environment. The family environment refers to the instrumental and emotional support provided by the family for physical activity. This was assessed using the *Family Support Scale*, which has been used in youth populations and showed a Cronbach alpha coefficient of 0.80 (7). In Morrissey et al. (7), the Family Support Scale was administered to adolescents; in our study, the version was adapted so who answered were the parent/guardian. The parent version was worded to reflect this change. Based on this, a parent/guardian reported how often, during *the last 7 days* they or another adult in the household: 1) watch their child participate in PA or sport (watch child do PA), 2) encourage their child to do sports or PA (encourage PA), 3) provide transport to a place where their child can do PA or sports (provide transportation), and 4) do PA or play sports with their child (do PA with child). The original instrument also included a positive reinforcement item (“told the child that is doing well in PA or sport”; family told), which was considered overlapping with family encouragement, so only the encouragement item was retained. Response options ranged from 1 (never) to 5 (every day). Scores for these four items were averaged to produce a composite score ranging from 1 to 5, with higher scores reflecting a more supportive family environment for PA. In our sample, the Cronbach alpha coefficient was 0.75.

The social environment was assessed through a *Home Neighborhood Environment questionnaire*. Perceived collective efficacy, as an indicator of social environment, was calculated as the average of social cohesion and social control scores. This instrument has been used in a previous multi-country study, which examined the association between collective efficacy and PA reporting Cronbach alpha coefficients of 0.81 (18). Social cohesion refers to the level of trust and attachment among neighbors. It was measured using five statements to which the parent/guardian indicated their agreement: 1) people around my neighborhood are willing to help their neighbors, 2) this is a close-knit neighborhood, 3) people in my neighborhood can be trusted, 4) people in my neighborhood generally do not get along with each other, and 5) people in my neighborhood do not share the same values, attitudes or beliefs. Response options ranged from 1 (strongly disagree) to 5 (strongly agree), with negatively worded items reverse-scored. The average of these five items produced a score ranging from 1 to 5, with higher scores indicating greater social cohesion. Social control reflects the perceived likelihood of neighborhood residents intervening in various scenarios This was measured using five items assessing the likelihood of intervention if: 1) children were skipping school and hanging out on the street corner, 2) children were spraying painting graffiti on a local building, 3) a child was showing disrespect to an adult, 4) there was a fight in front of their house, and 5) the fire station closest to their home was threatened with budget cuts. Responses ranged from 1 (very unlikely) to 5 (very likely). The average of these five items generated a score ranging from 1 to 5, with higher scores indicating stronger social control. The final collective efficacy score was obtained by averaging the scores for social cohesion and social control. Higher collective efficacy represents a more positive social environment. The Cronbach alpha coefficient in our sample for collective efficacy was 0.71.

The built environment was assessed using the *Neighborhood Environment Walkability Scale—Youth Version (NEWS-Y).* This instrument is an adaptation of the original NEWS, which was designed for adults, and was previously modified for use in studies of adolescents. In the NEWS-Y, parents/guardians report their perception of their neighborhood-built environment as it relates to their child. Details of the adaptation and psychometric properties have been provided in a previous study, which reported Cronbach alpha coefficients between 0.75 and 0.87 (28). For this study, eight items from the NEWS-Y survey were utilized: 1) land use diversity (e.g., distance to convenience stores), 2) proximity to recreation facilities (e.g., distance to indoor/outdoor recreation facilities), 3) land access (e.g., walking distance to stores or bus stop), 4) street connectivity (e.g., there are not many dead ends), 5) walk facilities (e.g., there are sidewalks), 6) aesthetic (e.g., there are interesting things to look at), 7) pedestrian safety (e.g., the traffic makes it difficult to walk), and 8) child safety (e.g., I'm afraid of my child being taken or hurt). Response options for items 1 and 2 were based on perceived distance to locations, rated on a Likert scale from 1 (1–5 min) to 5 (31+ min). For the remaining items, responses were scored on a four-point Likert scale ranging from 1 (strongly disagree) to 4 (strongly agree). Given the variation in response options across items, an overall physical environment score was created by calculating z-scores for each of the eight subscales and summing them. Higher z-scores indicate a more positive and walkable built environment. The Cronbach alpha coefficient in our sample was 0.68.

### 2.5 Analysis

The Shapiro–Wilk test was used to assess the distribution of continuous variables. Most variables were not normally distributed, except for accelerometer awake wear time and sedentary time which exhibited normal distributions. For uniformity, continuous data are reported as medians with interquartile ranges (IQRs). Categorical data are presented as frequencies and percentages. The General Linear Model (GLM) procedure in SPSS was employed to analyze the effects of family, social, and built environment on PA (MVPA, LIPA, and steps) and sedentary time as continuous measures. The model included several candidate variables considered based on prior literature (29–31). After preliminary descriptive and exploratory analyses, sex, race, and school calendar status (i.e., whether data collection occurred during the school year or vacation period) were retained as fixed factors. BMI and accelerometer awake wear time were included as covariates to adjust for individual differences in body weight status and measurement exposure. The build terms option in SPSS was employed to specify the model terms. The main effects of family, social, and built environment were included in the model to assess their individual contributions to the variance in the dependent variables. No interaction terms were included. Additionally, exploratory stratified analyses by sex were conducted. SPSS version 29 was used for the analysis. A *p*-value < 0.05 was considered statistically significant.

## 3 Results

### 3.1 General characteristics of the sample

The general characteristics of the sample are presented in Table 1. Participants wore accelerometers for an average of 14.4 h per day while awake. During this time, adolescents accumulated 27.7 min/day in MVPA, 3.8 h in LIPA, and an average of 6,896 steps per day, while spending 9.9 h in sedentary time. Notably, only 1 in 10 adolescents met the PA recommendation of ≥60 min of MVPA on average per day (27).

### 3.2 Description of family, social, and built environment

When examining the items within each environment (family, social, and built), the highest scores in the family environment were reported for emotional support (encouragement), while the lowest was for supervision support (watching the child do PA) and co-participation (do PA with the child). In the social environment or Collective Efficacy, the score for Social Cohesion (trust and attachment among neighbors) was lower than that for Social Control (likelihood of residents intervening in various scenarios). For the built environment, participants reported that it would take at least 10 min to walk to shops and recreational facilities, as reflected in low scores on the items of *land use diversity* and *proximity to recreational facilities* (Table 1). This aligns with the low score on the item of *access to land*, where participants expressed limited agreement on the availability of shops and public transport within walking distance. Lower scores were also observed for *aesthetics* (the neighborhood offers many interesting things to look at while walking) and child safety (children may face insecurity in various scenarios) (Table 1).

### 3.3 Associations between environments, physical activity, and sedentary time

The family environment was positively associated with MVPA, LIPA, and steps per day, and negatively associated with sedentary time. Specifically, each unit higher in family support was associated with approximately 5 additional minutes of MVPA, 8 additional minutes of LIPA, 688 more steps per day, and 13.2 fewer minutes of sedentary time per day. No significant associations were found between the overall social or built environment scores and PA or sedentary time (Table 2).

**Table 2:** Associations between family, social, and built environment with sedentary time and physical activity in adolescents (*n* = 308).

Variable	MVPA(min/day)				LIPA(min/day)				Steps/day				Sedentary time (min/day)			
	Beta	Lower	Upper	Sig	Beta	Lower	Upper	Sig	Beta	Lower	Upper	Sig	Beta	Lower	Upper	Sig
Family environment (score)	5.2	3.0	7.5	<0.01	7.9	2.0	13.9	0.01	688	396	979	<0.01	−13.2	−20.4	−5.9	<0.01
Social environment (score)	−2.1	−5.7	1.4	0.24	−8.7	−17.9	0.4	0.06	−259	−730	211	0.28	10.8	−0.4	22.1	0.06
Physical environment (z-score)	0.2	−0.5	0.9	0.56	−0.7	−2.5	1.0	0.41	12	−76	100	0.79	0.5	−1.6	2.6	0.63

Adjusted by age, sex, race, in/out-of-school status, wear time accelerometer, and BMI. LIPA, Light intensity physical activity; MVPA, Moderate-to-vigorous physical activity; PA, physical activity.

Regarding the associations between individual items of family, social, and built environment, most of the components of the family environment (*watch, encourage, and transport*) were associated with both PA (positive association) and sedentary time (negative associations), except for the component co-participation (*do PA with child*) (Table 3).

**Table 3:** Associations between subcomponents of family, social, and built environment with physical activity and sedentary time in adolescents (*n* = 308).

Variable	MVPA (min/day)				LIPA (min/day)				Steps/day				Sedentary time (min/day)			
	Beta	Lower	Upper	Sig	Beta	Lower	Upper	Sig	Beta	Lower	Upper	Sig	Beta	Lower	Upper	Sig
Family environment (score)	5.2	3.0	7.5	<0.01	7.9	2.0	13.9	0.01	688	396	979	<0.01	−13.2	−20.4	−5.9	<0.01
Watch child do PA	4.5	2.8	6.2	<0.01	7.1	2.6	11.5	0.00	646	428	863	<0.01	−11.5	−17.0	−6.1	<0.01
Encourage PA	2.1	0.6	3.7	0.01	4.6	0.5	8.7	0.03	282	76	488	0.01	−6.7	−11.7	−1.7	0.01
Provide transportation	3.4	1.8	5.0	<0.01	3.9	−0.3	8.1	0.07	393	187	600	<0.01	−7.3	−12.4	−2.2	0.01
Do PA with child	1.5	−0.7	3.7	0.18	1.6	−4.1	7.3	0.58	217	−71	504	0.14	−3.1	−10.1	3.9	0.39
Social environment^a^ (score)	−2.1	−5.7	1.4	0.24	−8.7	−17.9	0.4	0.06	−259	−730	211	0.28	10.8	−0.4	22.1	0.06
Social cohesion	−4.4	−7.4	−1.4	0.01	−6.2	−14.0	1.7	0.12	−547	−942	−152	0.01	10.6	0.9	20.2	0.03
Social control	1.0	−1.7	3.8	0.46	−5.5	−12.6	1.7	0.13	146.0	−220	512	0.43	4.4	−4.4	13.2	0.33
Built environment (z-score)	0.2	−0.5	0.9	0.56	−0.7	−2.5	1.0	0.41	12	−76	100	0.79	0.53	−1.62	2.68	0.63
Land use diversity	0.7	−1.1	2.5	0.44	2.5	−2.1	7.2	0.29	24.3	−208	257	0.84	−3.2	−8.9	2.5	0.27
Proximity to recreation facilities	0.1	−1.9	2.1	0.94	−2.2	−7.3	2.9	0.39	−35.9	−293	221	0.78	2.1	−4.1	8.4	0.50
Land access	−0.2	−2.3	1.9	0.84	−5.0	−10.3	0.3	0.07	−84	−352	184	0.54	5.2	−1.4	11.7	0.12
Street connectivity	1.0	−1.4	3.4	0.41	0.2	−6.1	6.5	0.95	133	−182	450	0.41	−1.2	−8.9	6.5	0.76
Walk facilities	0.3	−1.9	2.4	0.82	−1.3	−6.9	4.3	0.65	22	−257	301	0.88	1.0	−5.8	7.9	0.76
Aesthetic	0.7	−1.8	3.3	0.56	−3.4	−9.9	3.1	0.31	59	−268	386	0.72	2.6	−5.4	10.7	0.52
Pedestrian safety	1.3	−1.9	4.5	0.41	6.7	−1.5	14.9	0.11	341	−73	757	0.11	−8.0	−18.1	2.1	0.12
Child safety	−1.9	−4.7	1.0	0.20	−4.3	−11.6	3.0	0.25	−276	−647	94	0.14	6.1	−2.8	15.1	0.18

a Social environment or Collective efficacy adjusted by age, sex, race, in/out-of-school status, wear time accelerometer, and BMI. MVPA, moderate-to-vigorous physical activity; LIPA, light intensity physical activity; PA, physical activity.

Examining the items that form the social environment (collective efficacy), only social cohesion was negatively associated with MVPA (*β* = −4.4) and steps per day (*β* = −547) and positively associated with sedentary time (*β* = 10.6). Regarding the items composing the built environment, no associations were observed with sedentary time or PA.

When we stratified by sex, the same overall pattern was observed, with only minor variations. Within the family environment, MVPA was positively associated in boys and girls (*β* = + 5.1, *β* = + 4.7, respectively), similar for LIPA (*β* = 3.9 in boys, 10.1 in girls), and steps per day (*β* = 693 in boys, *β* = 601 in girls). Sedentary time was inversely associated in both sexes (*β* = −12.2 in boys, *β* = −15.6 in girls). In contrast, no significant associations were found for the social or built environment domains in either stratum (data not shown).

## 4 Discussion

### 4.1 Main findings

The main finding of this study is that, among the environmental factors examined, the family environment showed the most consistent associations with both PA and sedentary time. Specifically, family support was positively associated with MVPA, LIPA, and steps per day, while it was inversely associated with sedentary time. In contrast, both the social and built environments seem to have less influence on physical activity and sedentary time in our sample. These results highlight the key role of family support in promoting adolescent physical activity and reducing sedentary time, suggesting that family-based strategies represent an important opportunity to encourage active lifestyles.

### 4.2 Family environment, physical activity, and sedentary time

Other studies have highlighted the influence of family, particularly parents, on young people's physical activity. Our findings align with these reports, indicating that parental support plays a key role in promoting physical activity in both children and adolescents (9, 32, 33). When we examined the different types of family support, we found that only co-participation in PA, which requires greater parental involvement, was not associated with adolescent activity levels. A systematic review by Edwardson et al. (14) found that support requiring a high level of parental involvement is more influential for younger children aged 6–11 years than for adolescents aged 12–18 years. On the other hand, Henriksen et al. (32) found that all types of parental support were similarly associated with adolescents’ PA in Denmark, reporting no differences between boys and girls. In our study, stratified analyses by sex showed that the associations remained consistent across sexes. Some minor variations were observed, such as slightly higher MVPA in boys and greater sedentary time and LIPA in girls, but these nuances were modest and did not change the overall conclusions Additionally, a qualitative study by Abdelghaffar et al. (34) exploring barriers and facilitators to PA among adolescents highlighted that family support, regardless of its type, was recognized as a key facilitator of PA. While direct parental involvement appears to become less critical in adolescence, other forms of support, such as providing transportation or offering emotional encouragement, may continue to positively influence adolescents’ PA.

### 4.3 Social environment, physical activity, and sedentary time

Regarding collective efficacy, no associations were observed with PA or sedentary time either in the overall sample or when stratified by sex. This contrasts with findings from other studies, which have shown that neighborhoods with a more favorable collective efficacy are positively associated with PA in young populations (17). On the other hand, Sullivan et al. (18) in a multi-country study, reported that the association between collective efficacy and PA varied depending on a country's income level. While a positive association was observed in high-income countries, a null or inverse association was found in low-income countries. Specifically, Sullivan et al. (18) reported for the United States, a high-income country, only a marginal, non-statistically significant association between collective efficacy and PA (MVPA) (*β* = 1.54; 95% CI: −0.09, 3.17). This may be attributed to socioeconomic disparities within the U.S. While the country is high-income overall, significant inequalities in access to resources and opportunities persist. Adolescents in lower-income neighborhoods may face challenges that limit the impact of collective efficacy. Additionally, the high collective efficacy score (4 out of 5) in our study suggests that, despite positive collective efficacy, other factors (e.g., safety or autonomy) may reduce their influence on PA. Moreover, a previous review suggested that collective efficacy may more accurately capture a community's ability to address social issues and provide public services, rather than directly shaping individual health behaviors (35). Thus, even in communities with high collective efficacy, better resources and safer environments do not necessarily translate into increased PA.

However, when analyzing collective efficacy components separately—social cohesion and social control—social cohesion was unexpectedly negatively associated with PA and positively associated with sedentary time. Social control, in turn, did not show significant associations with either PA or sedentary time. Social cohesion refers to the strength of relationships among community members, including mutual support and shared values and beliefs. Rogers et al. (36) observed a positive association between social cohesion and PA and a negative association with sedentary time, suggesting that the sense of belonging fostered by social cohesion may increase the likelihood of engaging in positive health-related behaviors in adolescents. In our sample, however, social cohesion may manifest in ways that do not involve PA and instead promote sedentary time. For example, it is plausible that in this context these social interactions could take the form of spending time together and talking, participating in church-related activities, or preparing and eating meals together, although these specific behaviors were not directly assessed in our study. This suggests that, in our sample, social cohesion may foster social interactions that are more centered around sedentary activities rather than promoting PA. Consistent with previous studies, the absence of an association for social control with PA has generally been reported as null or negative (35). Moreover, it has been suggested that environments with greater social control may be more closely linked to the prevention of risky behaviors than to the promotion of PA (37). Future research should explore the specific social dynamics that influence these behaviors and examine whether interventions can leverage social cohesion, while also considering the more limited role of social control, to encourage more active lifestyles.

### 4.4 Built environment, physical activity, and sedentary time

Our results indicate no association between the built environment and either PA or sedentary time, either in the overall sample or when stratified by sex. While numerous studies have explored the influence of the built environment on PA, findings remain inconsistent. This variability could be explained by differences in methodologies (i.e., subjective vs. objective assessments) used to assess the built environment and PA. For instance, Dowda et al. (10) found a positive association between objectively measured environmental characteristics (using Geographic Information System, GIS) and PA assessed via accelerometry. Notably, these associations were stronger in adolescents than in children, likely due to adolescents’ greater independence. Similar findings had been previously reported in a systematic review (19). Conversely, Hinckson et al. (38) assessed the built environment using both objective GIS-based measures and adolescents’ perceptions. Their findings indicated that only the perceived built environment was associated with PA. In our study, the built environment was assessed based on parents’ perceptions. These perceptions may differ from those of adolescents and may reflect awareness rather than the actual availability of resources for PA. Additionally, various individual factors such as motivation or parents’ own PA levels can shape these perceptions. For instance, less motivated or less active parents may perceive fewer opportunities and be less aware of available resources compared to their more active counterparts. Therefore, the differences in measurement methods likely explain the discrepancies between our findings and those of previous studies. While objective measures are generally more reliable than self-reports, they capture different aspects of the built environment. Objective measures provide information on the availability of PA resources, but how individuals perceive these opportunities may have a distinct influence on behavior. A combined approach integrating both subjective and objective measures could offer a more comprehensive understanding of the relationship between the built environment and PA.

### 4.5 Limitations

This study measured PA across the full day using accelerometers. The family, social, and built environment may reflect, to a greater extent, contexts outside school hours, such as the home and neighborhood. While accelerometer data include time information, isolating PA outside school hours would require assumptions about individual schedules and routines that were not feasible within the scope of this study. We analyzed total daily activity, recognizing that this may not fully correspond to the timing of the environmental factors assessed. Nonetheless, the associations observed, especially those related to family support, remain relevant and suggest broader influences on adolescents’ PA patterns. Future studies could benefit from time-specific analyses to explore these relationships in more detail.

Additionally, some questionnaires were adapted and completed by parents rather than adolescents themselves, which may limit the extend to which these measures capture adolescents’ own perceptions and behaviors, and should be considered when interpreting the findings.

## 5 Conclusions

Overall, our findings emphasize the critical role of family support in shaping adolescents’ PA and the time spent in sedentary behaviors, while noting that we observed limited associations with the social and built environment, whose influences appear complex and context dependent. Future research should further explore these relationships using integrated methodological approaches to better inform interventions aimed at promoting active lifestyles in youth. From a practical perspective, our results highlight the relevance of family-based strategies within public health policies, while also underscoring the importance of creating supportive environments through broader community and urban planning initiatives.

## Data Availability

The raw data supporting the conclusions of this article will be made available by the authors, without undue reservation.

## References

[1] van Sluijs EMF Ekelund U Crochemore-Silva I Guthold R Ha A Lubans D Physical activity behaviours in adolescence: current evidence and opportunities for intervention. Lancet. (2021) 398(10298):429–42. 10.1016/S0140-6736(21)01259-9 34302767 PMC7612669

[2] Chaput JP Willumsen J Bull F Chou R Ekelund U Firth J 2020 WHO guidelines on physical activity and sedentary behaviour for children and adolescents aged 5–17 years: summary of the evidence. Int J Behav Nutr Phys Act. (2020) 17:1232–43. BioMed Central Ltd. 10.1186/s12966-020-01037-z PMC7691077 33239009

[3] Carson V Hunter S Kuzik N Gray CE Poitras VJ Chaput JP Systematic review of sedentary behaviour and health indicators in school-aged children and youth: an update. Appl Physiol Nutr Metab. (2016) 41:S240–65. Canadian Science Publishing. 10.1139/apnm-2015-0630 27306432

[4] Guthold R Stevens GA Riley LM Bull FC. Global trends in insufficient physical activity among adolescents: a pooled analysis of 298 population-based surveys with 1·6 million participants. Lancet Child Adolesc Health. (2020) 4(1):23–35. 10.1016/S2352-4642(19)30323-2 31761562 PMC6919336

[5] Sallis J Neville O. Ecological models of health behaviors. In: Glanz K Rimer B Viswanath K, editors. Health Behavior: Theory, Research, and Practice. San Francisco, CA: Fifth (2015). p. 66–87.

[6] Ding D Sallis JF Kerr J Lee S Rosenberg DE. Neighborhood environment and physical activity among youth: a review. Am J Prev Med. (2011) 41(4):442–55. 10.1016/j.amepre.2011.06.036 21961474

[7] Morrissey JL Janz KF Letuchy EM Francis SL Levy SM. The effect of family and friend support on physical activity through adolescence: a longitudinal study. Int J Behav Nutr Phys Act. (2015) 12(1). 10.1186/s12966-015-0265-6 26289232 PMC4545918

[8] Laird Y Fawkner S Kelly P McNamee L Niven A. The role of social support on physical activity behaviour in adolescent girls: a systematic review and meta-analysis. Int J Behav Nutr Phys Act. (2016) 13. BioMed Central Ltd. 10.1186/s12966-016-0405-7 27387328 PMC4937604

[9] Tandon P Grow HM Couch S Glanz K Sallis JF Frank LD Physical and social home environment in relation to children’s overall and home-based physical activity and sedentary time. Prev Med (Baltim). (2014) 66:39–44. 10.1016/j.ypmed.2014.05.019 PMC4125450 24887496

[10] Dowda M Dishman RK Saunders RP Pate RR. Associations between three measures of physical activity and selected influences on physical activity in youth transitioning from elementary to middle school. Sports Med Health Sci. (2021) 3(1):21–7. 10.1016/j.smhs.2021.02.004 35782676 PMC9219254

[11] Rhodes RE Perdew M Malli S. Correlates of parental support of child and youth physical activity: a systematic review. Int J Behav Med. (2020) 27:636–46. Springer. 10.1007/s12529-020-09909-1 32529629

[12] Yao CA Rhodes RE. Parental correlates in child and adolescent physical activity: a meta-analysis. Int J Behav Nutr Phys Act. (2015) 12. BioMed Central Ltd. 10.1186/s12966-015-0163-y PMC4363182 25890040

[13] Morrissey JL Wenthe PJ Letuchy EM Levy SM Janz KF. Specific types of family support and adolescent non-school physical activity levels. Pediatr Exerc Sci. (2012) 24(3):333–46. 10.1123/pes.24.3.333 22971551 PMC3442951

[14] Edwardson CL Gorely T. Parental influences on different types and intensities of physical activity in youth: a systematic review. Psychol Sport Exerc. (2010) 11:522–35. 10.1016/j.psychsport.2010.05.001

[15] Kepper MM Myers CA Denstel KD Hunter RF Guan W Broyles ST. The neighborhood social environment and physical activity: a systematic scoping review. Int J Behav Nutr Phys Act. (2019) 16. BioMed Central Ltd. 10.1186/s12966-019-0873-7 31815626 PMC6902518

[16] Suglia SF Shelton RC Hsiao A Wang YC Rundle A Link BG. Why the neighborhood social environment is critical in obesity prevention. J Urban Health. (2016) 93:206–12. Springer Science and Business Media Deutschland GmbH. 10.1007/s11524-015-0017-6 26780582 PMC4794461

[17] Franzini L Elliott MN Cuccaro P Schuster M Gilliland MJ Grunbaum JA Influences of physical and social neighborhood environments on children’s physical activity and obesity. Am J Public Health. (2009) 99(2):271–8. 10.2105/AJPH.2007.128702 19059864 PMC2622771

[18] Sullivan SM Broyles ST Barreira T V Chaput JP Fogelholm M Hu G Associations of neighborhood social environment attributes and physical activity among 9–11 year old children from 12 countries. Health Place. (2017) 46:183–91. 10.1016/j.healthplace.2017.05.013 28544991

[19] McGrath LJ Hopkins WG Hinckson EA. Associations of objectively measured built-environment attributes with youth moderate–vigorous physical activity: a systematic review and meta-analysis. Sports Med. (2015) 45:841–65. Springer International Publishing. 10.1007/s40279-015-0301-3 25618013

[20] An R Shen J Yang Q Yang Y. Impact of built environment on physical activity and obesity among children and adolescents in China: a narrative systematic review. J Sport Health Sci. (2019) 8:153–69. Elsevier B.V.. 10.1016/j.jshs.2018.11.003 30997262 PMC6451055

[21] Prins RG Oenema A van der Horst K Brug J. Objective and perceived availability of physical activity opportunities: differences in associations with physical activity behavior among urban adolescents. Int J Behav Nutr Phys Act. (2009) 6. 10.1186/1479-5868-6-70 19832969 PMC2770555

[22] Bejarano CM Carlson JA Cushing CC Kerr J Saelens BE Frank LD Neighborhood built environment associations with adolescents’ location-specific sedentary and screen time. Health Place. (2019) 56:147–54. 10.1016/j.healthplace.2019.01.015 30743089 PMC6477917

[23] Harris PA Taylor R Thielke R Payne J Gonzalez N Conde JG. Research electronic data capture (REDCap)-a metadata-driven methodology and workflow process for providing translational research informatics support. J Biomed Inform. (2009) 42(2):377–81. 10.1016/j.jbi.2008.08.010 18929686 PMC2700030

[24] Harris PA Taylor R Minor BL Elliott V Fernandez M O’Neal L The REDCap consortium: building an international community of software platform partners. J Biomed Inform. (2019) 95. Academic Press Inc. 10.1016/j.jbi.2019.103208 31078660 PMC7254481

[25] Tudor-Locke C Barreira T V Schuna JM Mire EF Katzmarzyk PT. Fully automated waist-worn accelerometer algorithm for detecting children’s sleep-period time separate from 24-h physical activity or sedentary behaviors. Appl Physiol Nutr Metab. (2014) 39(1):53–7. 10.1139/apnm-2013-0173 24383507

[26] Evenson KR Catellier DJ Gill K Ondrak KS McMurray RG. Calibration of two objective measures of physical activity for children. J Sports Sci. (2008) 26(14):1557–65. 10.1080/02640410802334196 18949660

[27] Bull FC Al-Ansari SS Biddle S Borodulin K Buman MP Cardon G World Health Organization 2020 guidelines on physical activity and sedentary behaviour. Br J Sports Med. (2020) 54(24):1451–62. 10.1136/bjsports-2020-102955 33239350 PMC7719906

[28] Rosenberg D Ding D Sallis JF Kerr J Norman GJ Durant N Neighborhood environment walkability scale for youth (NEWS-Y): reliability and relationship with physical activity. Prev Med (Baltim). (2009) 49(2–3):213–8. 10.1016/j.ypmed.2009.07.011 19632263

[29] Suárez-Reyes M Beyl RA Katzmarzyk PT Staiano AE. Joint associations of moderate-to-vigorous physical activity and sedentary time with adiposity and cardiometabolic risk factors in adolescents. Med Sci Sports Exerc. (2025) 57(7):1319–25. 10.1249/MSS.0000000000003663 39898606 PMC13069828

[30] Ferrari GdM Pires C Solé D Matsudo V Katzmarzyk PT Fisberg M. Factors associated with objectively measured total sedentary time and screen time in children aged 9–11 years. J Pediatr (Rio J). (2019) 95(1):94–105. 10.1016/j.jped.2017.12.003 29306718

[31] Eglitis E Miatke A Virgara R Machell A Olds T Richardson M Children’s health, wellbeing and academic outcomes over the summer holidays: a scoping review. Children. (2024) 11(3):287. 10.3390/children11030287 38539322 PMC10969660

[32] Henriksen PW Ingholt L Rasmussen M Holstein BE. Physical activity among adolescents: the role of various kinds of parental support. Scand J Med Sci Sports. (2016) 26(8):927–32. 10.1111/sms.12531 26346509

[33] Hosokawa R Fujimoto M Katsura T. Parental support for physical activity and children’s physical activities: a cross-sectional study. BMC Sports Sci Med Rehabil. (2023) 15(1). 10.1186/s13102-023-00700-9 37491297 PMC10367251

[34] Abdelghaffar EA Hicham EK Siham B Samira EF Youness EA. Perspectives of adolescents, parents, and teachers on barriers and facilitators of physical activity among school-age adolescents: a qualitative analysis. Environ Health Prev Med. (2019) 24(1). 10.1186/s12199-019-0775-y 30961543 PMC6454728

[35] Gao Z Chee CS Omar Dev RD Liu Y Gao J Li R Social capital and physical activity: a literature review up to march 2024. Front Public Health. (2025) 13. Frontiers Media SA. 10.3389/fpubh.2025.1467571 PMC11860974 40013056

[36] Rogers BJ Alphonso SR Neally SJ Deng Y Moniruzzaman M Tamura K. The role of the perceived neighborhood social environment on adolescent sedentary behavior and physical activity: findings from add health. J Community Health. (2024) 49(4):635–43. 10.1007/s10900-024-01332-x 38374312 PMC11407792

[37] Wang Y Chen M Lee JH. Adolescents’ social norms across family, peer, and school settings: linking social norm profiles to adolescent risky health behaviors. J Youth Adolesc. (2019) 48(5):935–48. 10.1007/s10964-019-00984-6 30715652 PMC6508974

[38] Hinckson E Cerin E Mavoa S Smith M Badland H Stewart T Associations of the perceived and objective neighborhood environment with physical activity and sedentary time in New Zealand adolescents. Int J Behav Nutr Phys Act. (2017) 14(1). 10.1186/s12966-017-0597-5 PMC5655834 29065897

